# Tips for teaching skin procedures to family medicine residents.

**DOI:** 10.12688/mep.20422.2

**Published:** 2025-12-12

**Authors:** Farhad Motamedi, Christine Rivet, Eric Wooltorton

**Affiliations:** 1 Department of Family Medicine, University of Ottawa, Ottawa, Ontario, Canada

**Keywords:** skin cancer, family medicine, training

## Abstract

**Background:**

As the incidence of skin cancer is growing and access to specialty care is becoming more limited in most settings, family physicians must take on an increasing role in detecting and managing skin cancer. Unfortunately, most family physicians are not adequately trained to perform the skin biopsies or excisions needed to do this.

**Aims and methods:**

Drawing on their own experience and the available literature, the authors present practical tips for clinical educators to teach skin procedures more effectively. Some of the tips are best applied in an academic centre with multiple learners; others are applicable to all settings.

**Conclusions:**

Family medicine training programs have a responsibility to ensure that their learners gain adequate expertise to diagnose skin cancers. This includes the ability to perform skin biopsies. These tips will help educators and institutions training family medicine residents design more effective ways to teach skin procedures.

## Background

With the growing incidence of skin cancer ^1^ the role of family physicians in detecting skin cancer has gained increasing importance. This is especially true in areas with poor access to specialists. Despite this, most family physicians are not adequately trained in skin cancer diagnosis and treatment ^2, 3^. Many educational interventions have been published to address this gap. Most are single day interventions, and while all focus on diagnosing skin cancer, many do not include teaching the skills to biopsy suspicious lesions ^4^.

Over the last fourteen years at out Family Medicine teaching site at the University of Ottawa, we have developed a bi-weekly procedures clinic where we teach skin cancer detection and management and provide opportunities to perform skin biopsies over a two-year curriculum ^5^. Through experience and feedback, we have adjusted the process over time and are now confident in our graduates’ skills in performing basic skin procedures, including elliptical excisions, punch biopsies, and shave biopsies. In this article we provide practical tips, learned through our experience, to help teach skin procedures to family medicine residents more effectively.

### Tip 1: Design a longitudinal curriculum

While most primary care residency programs offer episodic or opportunistic experiences for learning skin procedures, we have found repeated exposure over the course of the Family Medicine residency, as a longitudinal experience, very effective. This design would require a referral based “procedures clinic” where several patients with lesions suspicious for skin cancer are seen over a half or full day. Residents rotating through such clinics periodically throughout their residency, have an opportunity to see, do, repeat, and repeat basic skin procedures before they graduate. This enables them to encounter cases with varying complexities and potentially deal with complications in a safe and supervised setting, while they master specific procedures. Involving Family Medicine residents in dedicated procedures clinics has been shown to increase their level of confidence in performing procedures ^6^.

### Tip 2: Combine teaching skin procedures with teaching about skin cancer

The goal of a robust skin procedures curriculum for family medicine residents is to train family physicians who are comfortable performing biopsies of lesions suspicious for skin cancer. For this skill to be useful, they must first be able to recognize skin lesions which warrant biopsy. We have found that our procedures clinic is the optimal opportunity for teaching about skin cancer. We typically start each clinic with a 30-minute interactive case-based teaching session. The longitudinal nature of the experience gives us an opportunity to cover many topics during these sessions. The focus in the earlier part of the year is to improve their basic knowledge by covering topics such as melanoma, non-melanoma skin cancers, and skin cancer mimics. Later in the year, we consolidate learning by showing pictures of cases we have seen over the years and asking learners to give their tentative diagnosis and procedure of choice before we reveal the pathology. The scenario-based interactive teaching is followed by clinical experience and bedside teaching when we see the patients and perform procedures. This combination helps solidify learning and improve retention. Throughout our teaching sessions we also introduce dermoscopy pictures of melanoma and non-melanoma skin cancers, and their mimics. This is meant as a simple introduction to this tool which we also use as part of our bedside teaching prior to performing biopsies. The family physicians overseeing our procedures clinic have received additional training in dermoscopy, and we emphasize to learners that such training is needed before this tool can be used independently.

### Tip 3: Repeat and repeat

Our teaching sessions are based on a 1-year curriculum which we repeat over 2 years. Inevitably each learner misses a few sessions due to vacation or call. Repeating the same session the following year improves the chances of each learner being exposed to each topic at least once. Furthermore, we have found that repetition of our teaching even benefits the learners who did not miss the session in their first year. Consistent with Kern’s model ^7^ of curriculum design, curriculum maintenance (keeping the program vibrant by having repetition, reinforcement and increased sophistication of learning at different levels), we have found that learners at earlier and later stages of training seem to benefit from different parts of the same presentation. Additionally, they often forget some concepts which are more likely to be retained after repetition. In fact, in over a decade of following this design, we have never had a resident complain about redundancy.

### Tip 4: Emphasize non-technical aspects of procedures early and often

Learners tend to focus on the technical skills of procedures. In fact, the non-technical aspects seem to take longer to master and are just as important, and in some cases more important, to the overall success of the procedure. These non-technical skills can be divided in three phases: prior, during, and after the procedure ^8^. Prior to the procedure we emphasize answering the patients’ questions to minimize anxiety; obtaining a proper informed consent; preparing the equipment needed; preparing the patient by positioning and draping them appropriately; and optimizing the conditions by positioning the light, the exam table, and ourselves to maximize access, visibility, and comfort. During the procedure, we emphasize communication with the patient, and ways of dealing with expected or unexpected complications such as bleeding. After the procedure, we emphasize the importance of clear and patient-centered oral and written instructions about appropriate wound care and follow-up.

### Tip 5: Tailor exposure to learners’ level of experience

A positive learning experience in training has a significant impact on whether learners will choose to do procedures after graduation. A learner who is not given enough independence and responsibility will quickly become disengaged. On the other hand, too much independence and responsibility too soon can lead to a negative experience which will scar the learner and create additional anxiety. We try to gauge each learner’s experience, then assign rooms or teams, and procedures, intentionally based on that information.

### Tip 6: Combine learners of different level together

If you have the luxury of working with multiple learners, assign less experienced learners to work with more experienced ones when performing skin procedures. This layered learning ^9^ gives more senior learners an opportunity to teach, which they usually appreciate. Depending on your comfort with the senior learner’s competence, it can also free you up to supervise multiple rooms at the same time. It is also beneficial for junior learners to see the expertise of their senior colleagues as it increases their confidence in the curriculum and the fact that they will gain the required skills in time.

### Tip 7: Keep it simple

It is tempting to try to teach more advanced topics such as advanced suturing techniques for better cosmetic results. In our experience, to ensure that all learners achieve the more important and basic skills, it is best to focus on those. More advanced topics may be intimidating and confusing to some learners and may ultimately discourage them from performing biopsies. Having said that, we do introduce them to these topics without going in depth, so that they are aware of the benefits, and those with greater interest in skin procedures can learn these skills in other settings during or after their training. But we emphasize that these are not necessary for most diagnostic skin biopsies.

### Tip 8: Involve learners in the management of results

Due to the delay in receiving the pathology report, it can often be easier to deal with the results than to involve learners in managing the reports. However, this is an important step in preparing them for independent practice and should be emphasized. Choosing the appropriate next step once a skin cancer has been diagnosed can be challenging and should be learned. Forwarding the results to learners and asking them about further management is a good opportunity for teaching and for assessing their knowledge.

### Tip 9: Encourage gradual independence

Once observed competence has been achieved, encourage residents to perform skin procedures outside of a formal skin procedure clinic by booking patients they see with suspicious lesions in their own clinics for biopsy. This approach helps them develop further competence and debunks the idea that skin procedures should only be done in referral-based procedure clinics by those with special interest or expertise in this area.

### Tip 10: Train and mentor a more junior staff to collaborate with you

In our experience it is difficult to lead a skin procedure curriculum and clinic alone. Training and mentoring a more junior interested staff to help you has many benefits including succession planning, providing coverage when one is away, and the ability to get second opinions and discuss complex cases with a colleague. This approach also fosters growth and innovation in the design and delivery of the curriculum through sharing of ideas.

### Tip 11: Be ready to take over from the learner

There are times when it is in the patient and the learner’s best interest for the supervisor to take over from the learner and perform all or parts of a procedure themselves and teach by demonstrating the procedure. But it is important to avoid the temptation to do this too often and too quickly as it can be discouraging for the learner and limit their experience and learning.

### Tip 12: Make it fun

Most learners enjoy learning and performing procedures. To keep them interested and engaged, our role is to help alleviate their anxiety by preparing them appropriately and emphasizing positive feedback. Constructive feedback, while critical, should be tailored to level. For less experienced learners, we focus our feedback on the more basic concepts, saving feedback on more advanced details and concepts for later in their training. This ensures that the learners don’t get discouraged early, while remaining engaged and curious later as their expertise grows. 

## Conclusions

A well-designed procedures clinic can be an effective way of teaching family medicine residents to diagnose skin cancers and perform incisional and excisional biopsies. These skills will enable these future family physicians to detect skin cancer at a higher rate in their patients, reduce delay in diagnosis and decrease the burden on our specialist colleagues by selectively referring only those with confirmed cancer who need further treatment. This can also promote resident wellness as there is literature showing an association between physician satisfaction and performing procedures ^10^.

## Ethics and consent

Ethical approval and consent were not required.

## Data Availability

No data are associated with this article.
