# Supplementary material for: Tips for teaching skin procedures to family medicine residents
Source: MedEdPublish (2016). 2025 Dec 12;15:23. Originally published 2025 May 19. [Version 2] doi: 10.12688/mep.20422.2 (PMC12749556; doi:10.12688/mep.20422.2)
Supplement: Peer review history

# Reviewer report: Reviewer response for version 2

**DOI:** 10.21956/mep.22957.r44840

**Published:** 2025-12-30

**Authors:** Bronz W T

**Affiliations:** 1 Division of Dermatology, Department of Internal Medicine, School of Medicine, Walailak University, Nakhon Si Thammarat, Thailand

The authors revised the manuscript appropriately.

If evidence from practice is presented, are all the underlying source data available to ensure full reproducibility?

Yes

Is the topic of the practical tips discussed accurately in the context of the current literature

Yes

Are all factual statements correct and adequately supported by citations?

Yes

Are the conclusions drawn balanced and justified on the basis of the presented arguments?

Yes

Are arguments sufficiently supported by evidence from the published literature and/or the authors’ practice?

Yes

Reviewer Expertise:

Dermatologist

I confirm that I have read this submission and believe that I have an appropriate level of expertise to confirm that it is of an acceptable scientific standard.

# Reviewer report: Reviewer response for version 2

**DOI:** 10.21956/mep.22957.r44841

**Published:** 2025-12-30

**Authors:** Paweł Pietkiewic

**Affiliations:** 1 University Leonardo Da Vinci, Zug, Switzerland

no further comments.

If evidence from practice is presented, are all the underlying source data available to ensure full reproducibility?

Yes

Is the topic of the practical tips discussed accurately in the context of the current literature

Yes

Are all factual statements correct and adequately supported by citations?

Yes

Are the conclusions drawn balanced and justified on the basis of the presented arguments?

Yes

Are arguments sufficiently supported by evidence from the published literature and/or the authors’ practice?

Yes

Reviewer Expertise:

dermatoscopy training

I confirm that I have read this submission and believe that I have an appropriate level of expertise to confirm that it is of an acceptable scientific standard.

# Reviewer report: Reviewer response for version 2

**DOI:** 10.21956/mep.22957.r44859

**Published:** 2025-12-29

**Authors:** Tayseer M Mansour

**Affiliations:** 1 Medical Education, Suez Canal University Faculty of Medicine (Ringgold ID: 68833), Ismailia, Ismailia Governorate, Egypt; 2 Taibah University, Al Madinah, Saudi Arabia

This is an excellent and highly practical manuscript that provides valuable, experience-based guidance for medical educators. The authors, Motamedi et al., have distilled 14 years of experience from their longitudinal procedures clinic into twelve clear, actionable tips for teaching skin procedures to family medicine residents. The article is well-structured, concise, and addresses a recognized gap in postgraduate medical training. The format is ideal for busy clinical educators looking for practical strategies to implement in their own programs.

 The authors have also been responsive to previous peer reviews, as evidenced by the revisions in version 2 which clarify the types of biopsies taught and the role of dermoscopy in their clinic. The manuscript is a strong contribution to the medical education literature and serves as a useful roadmap for developing similar educational experiences.

If evidence from practice is presented, are all the underlying source data available to ensure full reproducibility?

Not applicable

Is the topic of the practical tips discussed accurately in the context of the current literature

Yes

Are all factual statements correct and adequately supported by citations?

Yes

Are the conclusions drawn balanced and justified on the basis of the presented arguments?

Yes

Are arguments sufficiently supported by evidence from the published literature and/or the authors’ practice?

Yes

Reviewer Expertise:

Medical education, curriculum development

I confirm that I have read this submission and believe that I have an appropriate level of expertise to confirm that it is of an acceptable scientific standard.

# Reviewer report: Reviewer response for version 1

**DOI:** 10.21956/mep.21854.r42272

**Published:** 2025-09-18

**Authors:** Bronz W T

**Affiliations:** 1 Division of Dermatology, Department of Internal Medicine, School of Medicine, Walailak University, Nakhon Si Thammarat, Thailand

The topic and content of the article are engaging and relevant. However, I would like to offer the following suggestions for improvement:

 1.    Involvement of Dermatologic Experts: The training program could benefit from the involvement of dermatology specialists, who can contribute their expertise through didactic or interactive lectures, spot diagnosis sessions, and instruction on dermoscopic findings, which are often more accurate than clinical inspection with the naked eye.

 2.    Dermatopathology Input: Collaboration with dermatopathologists may enhance the course by guiding residents in selecting appropriate biopsy sites, thereby reducing the risk of false-negative histopathologic results.

 3.    Specification of Biopsy Techniques: The authors may consider specifying which types of skin biopsies (e.g., shave, punch, incisional, or excisional) are included in the training curriculum.

 4.    Pre- and Post-Operative Care: It may be beneficial to include content on pre-operative evaluation and post-procedural care to ensure comprehensive procedural training.

If evidence from practice is presented, are all the underlying source data available to ensure full reproducibility?

Yes

Is the topic of the practical tips discussed accurately in the context of the current literature

Yes

Are all factual statements correct and adequately supported by citations?

Yes

Are the conclusions drawn balanced and justified on the basis of the presented arguments?

Yes

Are arguments sufficiently supported by evidence from the published literature and/or the authors’ practice?

Yes

Reviewer Expertise:

Dermatologist

I confirm that I have read this submission and believe that I have an appropriate level of expertise to confirm that it is of an acceptable scientific standard, however I have significant reservations, as outlined above.

# Response

**Published:** 2025-12-03

**Authors:** Farhad Motamedi

**Affiliations:** Family Medicine, University of Ottawa Faculty of Medicine, Ottawa, Ontario, Canada

Thank you for reviewing our manuscript, and for your helpful comments. We are submitting a revised version to specify the procedures we perform, as you suggested, and to add a comment about dermoscopy.   Our procedure clinic in led by 2 family physicians who have both completed the online Dermoscopy program through Cadfiff University.  One of them has also obtained a diploma in Dermatology through Cardiff University.  They have also spent time with the dermatopathologists at our hospital to learn about the best way to obtain the optimum biopsy samples, which they in turn convey to our learners.  However, they are not dermatologists.  Unfortunately, there is a lack of dermatologists in Canada.  For this reasons it is critical for our family medicine residents to learn the skills discussed in this manuscript.  For the same reason, it would be very difficult to involve a dermatologist in a longitudinal teaching program in most Canadian settings.  As discussed, our clinic occurs every two weeks throughout the year, so this would require a significant time commitment from a dermatologist.  Having said that, dermatologists often give lectures to family medicine residents in Canada, but these are done in a larger classroom setting and serve a different but complementary purpose to the longitudinal experience we are presenting here. As for your comment about teaching about pre and post operative care, the preoperative discussion occurs at bedside prior to performing the biopsies, and as mentioned in tip #3, we discuss post operative care verbally with the learners and patients and provide them written instruction on post care.  As this is repeated for every case, the learners become very familiar with it. We hope you will find this explanation helpful.  We would be thankful if you would take the time to review our revised version.

 Thank you again for your interest in this topic and in our work. F. Motamedi, MD

# Reviewer report: Reviewer response for version 1

**DOI:** 10.21956/mep.21854.r42024

**Published:** 2025-06-16

**Authors:** Paweł Pietkiewic

**Affiliations:** 1 University Leonardo Da Vinci, Zug, Switzerland

I have read the manuscript "Practical Tips" by Motamedi, Rivet, and Wooltorton with great interest. Drawing from their experience as Family Doctors and mentors, the authors offer insightful observations on gaps in medical training. These include limitations in curricula as well as non-theoretical aspects of clinical practice, such as shared responsibility, maintaining learner engagement, knowledge consolidation, and the development of stratified expertise and collegiality. They also highlight the value of incorporating fun into the learning process, a factor that is getting an increasing recognition.

 A central theme of the manuscript is the emphasis on simplicity, repetition and acceptance, all of which are crucial for effective teaching and long-term retention. The work presents a modern, learner-centered approach to medical education that will likely appeal to a wide audience of educators and clinical trainers.

 The manuscript is concise, logically organized, and written in clear English. I noted a single typo at the beginning of the second paragraph in the Background section ('out' instead of 'our'. Aside from this minor issue, I have no further concerns.

If evidence from practice is presented, are all the underlying source data available to ensure full reproducibility?

Yes

Is the topic of the practical tips discussed accurately in the context of the current literature

Yes

Are all factual statements correct and adequately supported by citations?

Yes

Are the conclusions drawn balanced and justified on the basis of the presented arguments?

Yes

Are arguments sufficiently supported by evidence from the published literature and/or the authors’ practice?

Yes

Reviewer Expertise:

dermatoscopy training

I confirm that I have read this submission and believe that I have an appropriate level of expertise to confirm that it is of an acceptable scientific standard.
